# Unknown Biases: Understanding How Anthropogenic Hybridization Impacts Genomic Diversity and Demographic Estimates

**DOI:** 10.3390/ani16152310

**Published:** 2026-07-27

**Authors:** Flor Hernández, Philip Lavretsky

**Affiliations:** Department of Biological Sciences, University of Texas at El Paso, El Paso, TX 79968, USA; fbh34@cornell.edu

**Keywords:** anthropogenic hybridization, domestication, demographic inferences, mallard

## Abstract

Understanding the genomic consequences of increasing anthropogenic hybridization between wild species and their domesticated forms requires careful consideration. The mallard (*Anas platyrhynchos*) and its sister species provide a unique system, as the worldwide release of domesticated game-farm mallards has resulted in widespread anthropogenic hybridization with wild mallards or endemic mallard-like congeners. While game-farm mallards continue to be released in mainland North America, they also gave rise to feral populations in Hawaii and New Zealand that interbred with endemic Hawaiian ducks (*Anas wyvilliana*) and New Zealand grey ducks (*Anas superciliosa superciliosa*), respectively. Here, we re-sequenced whole genomes representing domestic and wild congeners, as well as the resulting hybrids spanning a range of admixture levels. Wild genomes consistently harbored higher levels of genetic diversity and lower runs of homozygosity than domestic lineages. Among ecoregions, we recovered the strongest statistical correlation between ancestry and summary statistics for North America, where stocking continues. Conversely, in New Zealand and Hawaii, where stocking efforts largely ceased or are limited, we find an uncoupling of summary statistics, likely as a result of genetic drift and/or adaptive introgression. Finally, demographic inferences from domesticated or hybrid genomes produced biased estimates of effective population size (N_E_) and divergence times, suggesting careful consideration when attempting to infer such histories with inbred and/or admixed individuals.

## Simple Summary

The frequency of anthropogenic hybridization is increasing worldwide, particularly because of the direct release of captively bred individuals. Such interactions are especially concerning as they can acutely lower the natural genetic variation and adaptability of wild populations. To understand such consequences, we compared genomic diversity estimates of released and feral populations of game-farm mallards, endemic congeners, and resulting hybrids in mainland North America, Hawaii, and New Zealand. Generally, the wild congeners in each ecoregion harbored the greatest genetic variety and the fewest signs of inbreeding compared to feral or hybrid individuals. We also demonstrate how captive-breeding and hybridization create substantial biases when attempting to reconstruct a species’ history. Together, we shed light on the genomic consequences of hybridization between wild and domestic animals, and the importance of understanding an animal’s ancestral background before drawing demographic conclusions.

## 1. Introduction

Anthropogenic hybridization refers to the process whereby human activities, directly or indirectly, lead to interbreeding between previously isolated taxa [1,2]. The frequency of such events has increased because of human-mediated habitat modifications and/or the introduction of exotic species that facilitate the breakdown of reproductive isolation between sympatric species [3] or result in secondary contact between previously allopatric taxa [1,4]. In recent decades, studies have highlighted numerous examples of anthropogenic hybridization occurring across a range of taxa, from plants [5,6] to mammals [7,8] and birds [9,10,11]. Among such interactions, the uncontrolled diffusion of domestic organisms, including establishment of feral populations, may not only introduce genes favored under artificial selection that are maladaptive in natural environments, but can also result in the disruption of locally adaptive gene complexes, both leading to a reduction in the fitness of wild populations [12].

The mallard (*Anas platyrhynchos*) represents one of the last successful domestication events, leading to the generation of dozens of domestic breeds today [11,13,14]. Among these breeds, the game-farm mallard was a product of captive breeding started in 1630s England for game stocking purposes [11]. Although considered to have minimal impacts on wild populations, recent landscape genomic analyses have revealed that it is the worldwide release of game-farm mallards that has resulted in widespread hybridization in all instances, including the formation of hybrid swarms on Hawaii and New Zealand through interaction with endemic Hawaiian ducks (*Anas wyviliana*; [15]) and New Zealand grey ducks (*Anas supercilious*; [16]), respectively. Similar scenarios are playing out in Eurasia [14,17] and North America [11], where continued game-farm mallard releases are resulting in expanding game-farm × wild hybrid populations, including large parts now considered hybrid swarms themselves [11,18]. Whereas these examples highlight the profound impact that human activities can have on wild populations, they also present an opportunity to test whether hybridization results in similar consequences, as each instance is between the same domestic lineage [11].

Here, we aim to explore the consequences of secondary contact between domestic and wild congeners on genomic diversity and demographic estimates using regions that exhibit diverse ecologies and differing degrees of domesticated mallard release, including Hawaii, New Zealand, and North America. We analyzed whole-genome sequences of genetically vetted domestic and wild parental species, as well as various backcrosses that comprise identified domestic × wild swarms in the three regions. The domestication process often profoundly alters the genomic landscape towards increasingly lowered levels of genetic diversity as these breeds are exposed to serial generations of artificial selection, whether intended or not [2,12]. As a result, we predict that genomes with an increasing proportion of domestic lineages will have lower levels of genetic diversity, increased runs of homozygosity, and generally higher levels of inbreeding as compared to their local wild relative [19,20]. Next, we hypothesize that the same measures of diversity/inbreeding will scale with the domestic level of admixture among hybrid offspring. Finally, introgressed domestic genes can result in complex genetic patterns that may obscure demographic signals and bias population genetic analyses [21,22,23]. Despite the advances of genome-sequencing and demographic models such as Pairwise Sequentially Markovian Coalescent (PSMC, [24]), few studies have explored how introgressive hybridization may affect PSMC inferences. In particular, analyzing genomes of hybrids that vary in admixed ancestry is required to fully realize the power of PSMC when attempting to reconstruct population histories from confounded genomes. Thus, we also take this opportunity to determine levels of bias when estimating effective population size (N_E_), often a critical value used to assess conservation status [23], using genomes of domestic or hybrid origin. Together, our study provides insight into the genetic consequences of anthropogenic hybridization and its impact on the overall genetic diversity of wild populations, something that is increasingly needed when attempting to conserve biodiversity in the Anthropocene [2,25].

## 2. Materials and Methods

### 2.1. Sampling, DNA Extraction, Whole-Genome Resequencing, and Variant Calling

A total of 18 muscle tissue samples, representing wild and domestic duck populations found in the Hawaiian Islands, New Zealand, and North America, were collected or provided by museums (Table 1). Note that we used ancestry assignments obtained from previous population genetics studies using partial-genome data conducted in Hawaii [15], New Zealand [16], and North America [11], as our sampling scheme did not permit direct assessment of ancestry from the genomes.

First, we downloaded raw Illumina sequences for recently published reference genomes for the Hawaiian duck ([26]; NCBI SRA BioSample SAMN57184893) and North American wild mallard ([22]; NCBI SRA BioSample SAMN36329575). For the remaining 16 tissues, DNA was extracted using a QIAGEN Blood & Cell Culture DNA kit according to the manufacturer’s protocols (Qiagen, Hilden, Germany), followed by additional cleaning using the Clean and Concentrator^TM^-25 kit as per the manufacturer’s instructions (Zymo Research, Irvine, CA, USA). Subsequently, DNA concentration and quality were determined using a Qubit fluorometer v 2.0 (Life Technologies, Carlsbad, CA, USA) and a NanoDrop spectrophotometer (Nanodrop 2000, Thermo Scientific, Waltham, MA, USA), respectively. Extracted DNA was then sent to Novogenetics LTD (Sacramento, CA, USA) for library preparation and sequencing using 150 bp PE chemistry on a NovaSeq 6000 (Illumina, San Diego, CA, USA) until ≥30 Gb was achieved per sample.

Raw Illumina paired-end reads were first merged with the program PEAR v.0.9.11 [27]. Then, we trimmed or discarded poor-quality sequences using Trimmomatic [28], followed by sequence alignment to the recently published reference-scale wild North American mallard genome ([22]; NCBI SRA BioSample SAMN36329575) using the Burrows Wheeler Aligner v. 07.15 (bwa [29]). Samples were then sorted and indexed in Samtools v1.6 [30], combined, and genotyped using the bcftools “mpileup” and “call” functions with the following parameters: “-c -A -Q 30 -q 30”, which set a base pair and overall sequence PHRED score of ≥30 to ensure that only high-quality sequences were retained. The resulting VCF file was then filtered using VCFtools v.0.1.17 [31] with a minimum quality of 30 (-minQ 30), a minimum depth of 10 (-minDP 10), and by removing all sites with a minimum allele depth of 5 (-remove-filtered ‘AD < 5’). These conservative thresholds were applied uniformly across genomes, guarding against false heterozygous genotypes that would otherwise inflate π and distort ROH and PSMC inference. Note that bioinformatic steps were automated using an in-house Python 3 script (Python scripts available at https://github.com/jonmohl/PopGen accessed on 1 March 2024; [18]).

### 2.2. Measures of Genetic Diversity

For each genome, we calculated nucleotide diversity (π) across non-overlapping 100-Kb windows using the custom script popgenWindows.py script from [32] (available at https://github.com/simonhmartin/genomics_general accessed on 29 March 2024). For runs of homozygosity (ROH), we used VCFtools v0.1.17 [31] and calculated all possible base-pairs. Finally, we also calculated per-individual inbreeding coefficients based on the F_HBD_ parameter as calculated in the RZooRoH R package v0.3.1 [33,34,35]. Briefly, RZooRoH implements hidden Markov models (HMM) to directly infer homozygous-by-descent (HBD) segments by considering the distance between two markers, as well as mutation and recombination rates, if available, to estimate an individual’s autozygosity (i.e., inbreeding coefficients; [33,34,35]). Here, we used the genome size of the reference mallard genome (~1.05 Gb; [22]), a recombination rate calculated for domestic mallards of 2 cM/Mb [36], a minimum allele frequency of 0.10 to exclude singleton SNPs, and 0% missingness. Note that while RZooRoH can bin HBD segments into different generations (i.e., recent versus ancestral inbreeding), we only extracted the overall per-sample F_HBD_ estimate.

### 2.3. Inference of Demographic History

We inferred effective population size (N_E_) across time for each genome using the Pairwise Sequential Markovian Coalescent (PSMC) model [24]. PSMC analyses require a consensus genome sequence (FASTQ) generated using SAMtools v1.3.1 [30], bcftools v1.1 [37], and the vcftutils.pl script from bcftools to call variants from the alignment with the following command: “samtools mpileup -C50 -uf ref.fas aln.bam | bcftools call -c | vcfutils.pl vcf2fq -d 10 -D 100 > diploid.fq”. The minimum depth (d) and maximum depth (D) were set to approximately a third and twice the average read depth of each genome, respectively, as recommended (also see https://github.com/lh3/psmc accessed on 18 March 2024). Parameters were set to include N = 30, t = 5, r = 5 and *p* = “4 + 30 × 2 + 4 + 6 + 10”, and analyses were run with 100 bootstrap replicates [26,38]. The generation time (G) was calculated for each duck based on its average maximum age at sexual maturity (*α*) and adult survival (*s*) following the formula G = *α* + (*s*/(1 − *s*)), and using a mutation rate of 1.0 × 10^−9^ per site per generation [18]. For mallard-like ducks, the age of maturity is typically 1 year (i.e., α = 1; [39]). However, we estimated the average survival rate individually. For wild mallards, this survival rate is ~0.57 (i.e., range: 0.46–0.68; [40,41,42]), resulting in an estimated generation time of 2.32 years. For the New Zealand grey duck, the survival rate is 0.56 [43], leading to an estimated generation time of 2.27 years. For Hawaiian ducks, the survival rate is 0.74 [44], resulting in an estimated generation time of 3.97 years. For all the domestic mallards and domestic × wild hybrids, we used the same estimated generation time as for wild mallards.

## 3. Results

All genomes were sequenced to mean coverage depths ranging from 17x–84x, with high alignment similarity (~99%) across 879,691,675 quality base pairs, including 63,805,135 single-nucleotide polymorphisms (SNPs) that passed filtering (Table 1).

### 3.1. Measures of Genetic Diversity

**Figure 1:**
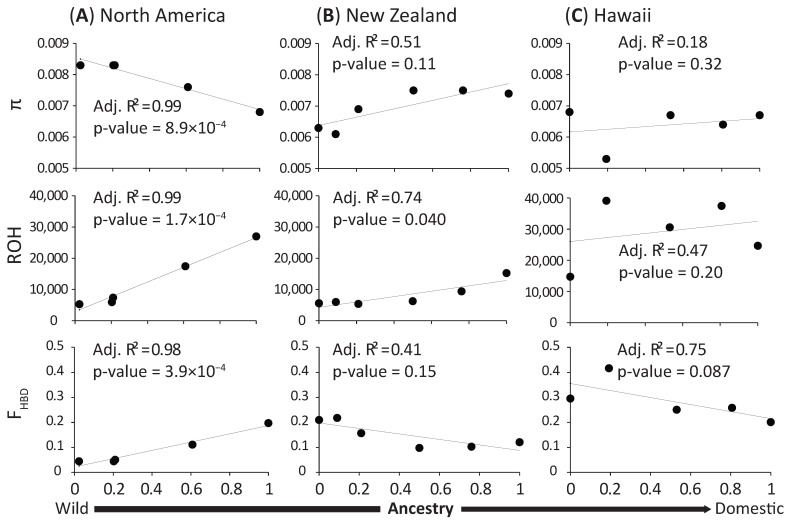
Correlation between the amount of respective domestic parental ancestry and per-sample measures of genetic diversity, calculated in 100 Kbp windows, for nucleotide diversity and runs of homozygosity, and with individual inbreeding coefficients (F_HBD_) based on all possible bi-allelic SNPs for samples collected in (**A**) mainland North America, (**B**) New Zealand, and (**C**) Hawaii. Respective regressions (adjusted R^2^) and *p*-values are provided, with significance considered at the 0.05 level.

Among genomes representing wild species, mallards had the highest nucleotide diversity (Avg. π = 0.0083), the smallest inbreeding coefficient (F_HBD_ = 0.044) and the smallest ROH (Avg. Autosomal ROH = 5306 bp) compared to Hawaiian ducks (Avg. π = 0.0068, F_HBD_ = 0.30, Avg. Autosomal ROH = 24,642 bp) and New Zealand grey ducks (Avg. π = 0.0063, F_HBD_ = 0.21, Avg. Autosomal ROH = 5582 bp) (Table 1). In each case, however, summary statistics for the representative domestic genomes were not necessarily worse compared to their respective endemic wild congeners. Those from Hawaii (Avg. π = 0.0067, Avg. F_HBD_ = 0.31, Avg. Autosomal ROH = 37,389 bp) generally had less favorable statistics compared to New Zealand (Avg. π = 0.0074, Avg. F_HBD_ = 0.14, Avg. Autosomal ROH = 15,255 bp) and North America (Avg. π = 0.0068, Avg. F_HBD_ = 0.068, Avg. Autosomal ROH = 26,991 bp). In fact, genomes representing game-farm × wild mallard hybrids in North America were the only group showing the expected and statistically significant correlations between domestic ancestry levels and summary statistics, where ROH and F_HBD_ were positively correlated and nucleotide diversity negatively correlated (Figure 1A). Conversely, we found positive relationships for nucleotide diversity and ROH, as well as a negative relationship for F_HBD_ against domestic ancestry in the New Zealand and Hawaii datasets. However, with the exception of ROH in the New Zealand dataset, relationships between levels of domestic ancestry and summary statistics were not statistically significant for genomes from New Zealand (Figure 1B; Table 1) or Hawaii (Figure 1C; Table 1).

### 3.2. Demographic History

**Figure 2:**
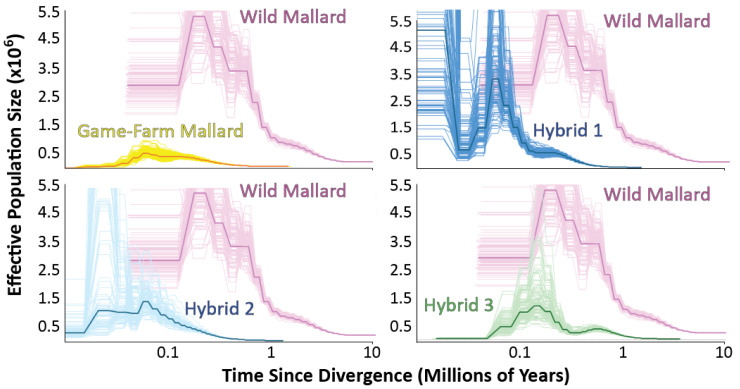
PSMC results for wild and domestic mallards and their hybrids from North America. In each analysis, the dark line denotes the average, while the lighter lines indicate the bootstraps.

**Figure 3:**
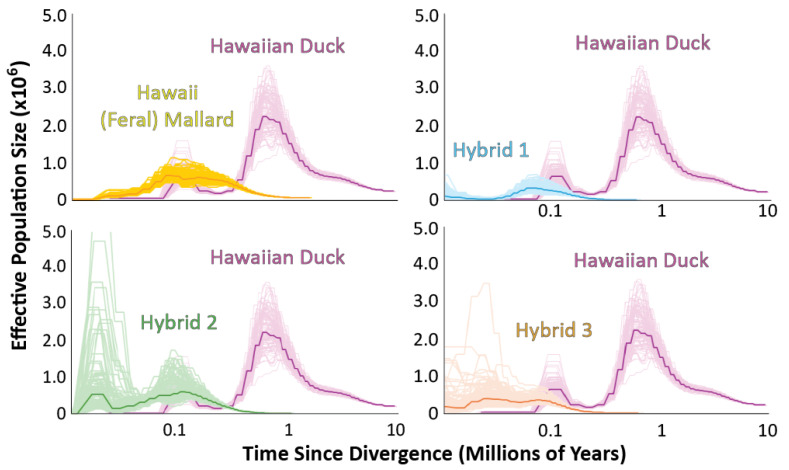
PSMC results for Hawaiian ducks, domestic mallards, and their hybrids from Hawaii. In each analysis, the dark line denotes the average, while the lighter lines indicate the bootstraps.

**Figure 4:**
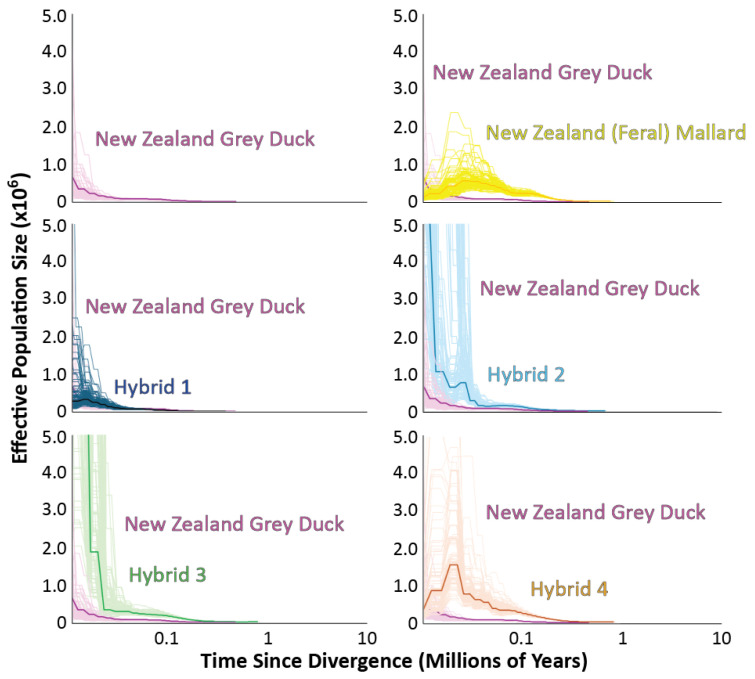
PSMC results for the New Zealand grey duck, domestic mallard, and their hybrids from New Zealand. In each analysis, the dark line denotes the average, while the lighter lines indicate the bootstraps.

PSMC analyses reliably estimated effective population size (N_E_) up to at least 1.5 million years before present (Figure 2, Figure 3 and Figure 4). Among genomes representing wild species, both the wild North American mallard and the Hawaiian duck exhibit similar demographic patterns, with an increasing effective population size starting around 1 million years before present (YBP). The wild North American mallard reached its peak effective population size around 175,000 YBP before declining and stabilizing at an effective population size of ~3 million since 100,000 YBP. In contrast, the Hawaiian duck reached its peak effective population size around 570,000 YBP before experiencing declines, followed by a second increase around 100,000 YBP, and stabilizing at a contemporary effective population size of around 50,000 individuals. Finally, we recovered a continuous increase in effective population size since around 150,000 YBP for the New Zealand grey duck, reaching a contemporary effective population size of 100,000.

Next, the general trend of increasing N_E_ in deep time, followed by a continuous reduction to near-zero today, which was recovered across genomes representing domestic lineages, is concordant with the fact that both Hawaii’s and New Zealand’s feral populations and those that are being released in North America are genetic lineages of the same game-farm mallard breed [11]. Nevertheless, the peak population sizes and times associated with changing N_E_ differed. Nearly identical demographic histories were recovered for domestic genomes from North America (Figure 2) and Hawaii (Figure 3), with N_E_ exponentially increasing up to ~700,000 years before present (YBP) before gradually declining to a contemporary effective population size of 10,000 individuals today. For the domestic genome from New Zealand, we recovered an increasing N_E_ to around 270,000 YBP before gradually decreasing to a contemporary effective population size of 230,000 today.

Finally, genomes representing the various domestic × wild hybrids tended to recover demographic histories closely tied to the parental taxa that are overwhelmingly represented in their genome. Although hybrid genomes did tend to show intermediate estimates in N_E_ across time relative to their parental taxa, both N_E_ and time were substantially distorted (Figure 2, Figure 3 and Figure 4). For example, genomes comprising more wild mallard in North America (i.e., Hybrid 1 & 2; Figure 2) showed a similar, though depressed, pattern in N_E_, but with these patterns consistently pushed forward in time. Interestingly, Hybrid 3, which was closest to a 50:50 ancestry split between game-farm and wild mallard based on partial genome data (Table 1; [11]), showed the expected combination effect by essentially depressing the wild signature through time but without a time-shift in those patterns (Figure 2). Conversely, genomes from Hawaii simply showed depressed estimates of N_E_, but all patterns were pushed forward in time (Figure 3). Finally, hybrid genomes from New Zealand generally followed the exponential increase in N_E_ through time as seen in the New Zealand grey duck (Figure 4).

## 4. Discussion

In addition to strong artificial selection [45,46,47], domestication and breed selection innately result in sequential bottlenecking, both resulting in the increasing chance of inheriting identical-by-descent genetic material [48]. Together, these pressures tend to exacerbate genetic diversity loss, increasing inbreeding depression, and resulting in extended homozygous regions in the offspring’s genome (i.e., increasing ROH) [48,49,50]. Indeed, we recovered the expected summary statistics and depressed effective population size estimates [51] for the genome representing the game-farm mallard that is currently being bred for release in North America and Eurasia (Table 1; Figure 1 and Figure 2). Importantly, we demonstrate that the genomes of the resulting hybrids between wild × game-farm pairings appear to be artificially reducing standing genetic variation in wild populations compared to what would be expected from wild × wild pairings (Figure 1A; Table 1). These genomic changes have recently been associated with morphological traits in which game-farm mallards tended to possess shorter, wider, and taller bills than their wild counterparts, which could influence their foraging behavior and diet preferences in the wild [47,52]. Additionally, recent studies have found a statistically significant but negative correlation between increasing game-farm mallard ancestry and migratory and breeding behaviors [53,54]. The movement of such traits that are likely maladaptive in the wild may contribute to the ever-declining wild mallard populations of North America, where game-farm mallard releases are highest [55,56]. Future work will benefit from extensive genomic sampling of individuals representing the spectrum of game-farm × wild mallard hybrids found in North America to test whether the diversity statistics remain correlated (Figure 1), while also directly linking domestically derived genomic variation to phenotype and fitness in the wild.

Interestingly, genomes representing populations on Hawaii and New Zealand provided uniquely different patterns, and unlike those recovered in North America (Table 1; Figure 1). We acknowledge that the per-region sample sizes potentially limited statistical power to detect weak correlations in New Zealand and Hawaii, and non-significance should not be read as evidence of no relationship. Nevertheless, we posit that the differing supplementation and/or the endemic congener histories with which introduced game-farm mallards interacted likely explains the somewhat unexpected genetic patterns (Table 1; Figure 1). Specifically, despite the same game-farm mallard source used in initial introductions, self-sustaining feral populations existing in Hawaii and New Zealand today have themselves been transformed [11,57,58,59], with the decoupling of summary statistics and levels of domestic ancestry consistent with genetic drift and/or adaptive introgression (Figure 1; Table 1). Formally testing between these scenarios will require denser within-region sampling and targeted analyses, including outlier-based analyses.

### Domestic and Hybrid Genomes Bias Demographic Inferences

Reconstructing demographic histories and assessing contemporary levels of effective population size from genomic data has become integral in evolutionary and conservation genetic studies [60,61,62]. Not only are such analyses helpful for understanding whether current standing genetic variation is a result of evolutionary or more contemporary trends, but establishing today’s effective population size is critical and often an important consideration when determining conservation priorities [63,64]. Given that PSMC analyses that provide such information use a single genome, what that genome represents is equally important [38,65,66]. To this end, we provide evidence that genomes subjected to the domestication process or of hybrid origins substantially bias demographic inferences (Figure 2, Figure 3 and Figure 4; [21,23,67]). First, our results confirm that the domestication process, which results in increasingly inbred lines (Table 1), causes demographic histories to be generally depressed, with similar patterns of slightly increasing N_E_ in deep time followed by decreasing trends in N_E_ to more recent times (Figure 2, Figure 3 and Figure 4). Next, whereas we recovered expected trends for genomes of hybrid individuals that followed demographics of their wild counterparts but with depressed values of N_E_, we unexpectedly also found that these trends were shifted forward in time. Thus, not only do inbred or hybrid genomes pose a serious consequence in estimating contemporary levels of N_E_, but any inferences about divergence time between lineages are equally biased [24,68,69]. This is especially concerning for genomes representing species but constructed from individuals born in captive settings (e.g., zoos) where unintentional selective pressures, potential bottlenecking, and even admixture may cause such genomes to significantly diverge from the original wild source. Together, researchers should always consider the individual’s source prior to making an effort to build genomes and reconstruct demographic histories, especially if such information is to be used to infer evolutionary histories or conservation planning.

## 5. Conclusions

Our comparative analysis shows that anthropogenic hybridization due to the direct release of captive-bred or domestic individuals can reshape the genomic diversity and demographic histories of wild populations. Of course, the ultimate outcome depends on regional history and ecology. In mainland North America, where game-farm releases are ongoing, the combination of an outbred and inbred genome results in hybrids with expected reductions in genomic diversity and increasing inbreeding. Conversely, in Hawaii and New Zealand, where releases have largely ceased, these relationships are decoupled, likely through genetic drift and adaptive introgression, respectively. Critically, we demonstrate that genomes of domesticated or hybrid origin systematically bias demographic reconstructions, depressing estimates of effective population size (N_E_) and distorting inferred divergence times. Because such inferences rest on a single genome, values drawn from inbred, captive-bred, or admixed individuals can substantially misrepresent a species’ true past. We therefore recommend that the ancestral history of each genome be carefully vetted before it is used to estimate diversity, effective population size, or divergence; particularly when these values inform conservation status or management. More broadly, our findings underscore both the erosive genomic consequences of releasing domesticated animals into the wild and the importance of interpreting those consequences within the specific ecological context in which hybridization occurs.

## Figures and Tables

**Table 1:** Per-sample metadata and genomic sequencing information. Per-sample average nucleotide diversity (π), runs of homozygosity (ROH), and inbreeding coefficients (FHBD), as well as the effective population size (NE) obtained from the most recent time estimate from demographic analyses. Note that samples are categorized by ecoregion and include a representative category based on the proportion of domestic ancestry assigned from partial genome data from previous population genetics studies in mainland North America [11], Hawaii [15], and New Zealand [16].

Sample.ID	Category	Ecoregion	State/Island/ Province	Latitude	Longitude	Collection Date	Sex	Age	No. Reads	Covered Bases (Mbp)	Alignment (%)	Mean Coverage (x)	Anc.Call	Domestic Parent Ancestry	Wild Parent Ancestry	F_HBD_	π	ROH	Ne	SRA BioProject	SRA Sample ID
MALLpl112316	Wild mallard	North America	New Mexico	32.953968	−107.29599	23-Nov-16	Male	Juvenile	20,777,591	34.07	99.31	67.4	Wild Mallard	0.024	0.98	0.044	0.0083	5306	2,900,000	PRJNA991977	SAMN36329575
CT44	Hybrid 1	North America	Connecticut	41.83671	−72.55994	31-Aug-18	Male	Juvenile	6,960,977	35.57	99.15	26.7	FX-GFM × WMA hybrid	0.20	0.80	0.044	0.0083	5939	4,780,000	PRJNA1499709	SAMN35641443
HHM154	Hybrid 2	North America	Massachusetts	42.375	−71.375	6-Aug-18	Male	Juvenile	6,876,137	35.57	99.15	26.9	FX-GFM × WMA hybrid	0.21	0.79	0.05	0.0083	7378	320,000	PRJNA1499709	SAMN35641613
NJ16	Hybrid 3	North America	New Jersey	39.8472	−75.0005	16-Aug-18	Male	Adult	5,673,930	35.56	99.12	21.9	F1-GFM × WMA hybrid	0.61	0.39	0.11	0.0076	17,444	35,000	PRJNA1499709	SAMN35641869
JIBNJ006	Game-farm mallard	North America	New Jersey	40.051487	−74.504385	2-Dec-17	Male	Juvenile	7,825,476	35.51	99.04	33	Game-farm Mallard	1	0.00	0.2	0.0068	26,991	15,000	PRJNA1499709	SAMN13391148
PL8002	New Zealand Grey Duck	New Zealand	West Coast	−42.3125	171.602333	14-May-18	Male	Adult	4,873,445	34	99.07	20.4	New Zealand grey duck	0.006	0.99	0.21	0.0063	5582	700,000	PRJNA1499709	SAMN41130506
PL8176	Hybrid 1	New Zealand	West Coast	−42.840819	171.02014	5-May-18	Male	Adult	5,258,468	35.54	99.03	20.9	FX- New Zealand grey duck Hybrid	0.11	0.89	0.22	0.0061	5982	280,000	PRJNA1499709	SAMN47463044
PL8032	Hybrid 2	New Zealand	Tasman	−41.411411	173.016067	5-May-18	Male	Adult	5,379,820	35.54	99.09	22.1	F2-New Zealand grey duck hybrid	0.22	0.78	0.16	0.0069	5359	1,070,000	PRJNA1499709	SAMN47462964
PL8023	Hybrid 3	New Zealand	Tasman	−41.795741	172.330897	5-May-18	Male	Juvenile	5,189,987	35.55	99.1	21.4	F1-New Zealand grey duck × feral New Zealand mallard	0.50	0.50	0.098	0.0075	6271	1,890,000	PRJNA1499709	SAMN47463030
PL8228	Hybrid 4	New Zealand	West Coast	−42.796218	170.918425	6-May-18	Male	Adult	5,338,833	34.74	99.11	22.6	F2-New Zealand mallard hybrid	0.75	0.25	0.1	0.0075	9396	880,000	PRJNA1499709	SAMN47462962
PL8109	New Zealand mallard	New Zealand	West Coast	−44.096992	171.4691	5-May-18	Male	Juvenile	5,435,162	35.55	99.1	21.2	New Zealand feral mallard	0.98	0.019	0.12	0.0074	15,255	230,000	PRJNA1499709	SAMN35662540
HAWD25386-4	Hawaiian duck	Hawaii	Kauai	22.03616	−159.77894	14-Sep-16	Male	Adult	22,332,019	34.02	99.1	84.6	Hawaiian duck	0	1	0.3	0.0068	14,734	50,000	PRJNA1451198	SAMN57184893
s169764410	Hybrid 1	Hawaii	Oahu	21.29399	−157.708943	17-Oct-03	Male	Adult	4,809,866	35.52	99	18.2	FX- Hawaiian duck Hybrid	0.19	0.81	0.42	0.0053	39,071	147,000	PRJNA1499709	SAMN13029883
AEJR4535	Hybrid 2	Hawaii	Oahu	21.368323	−157.710297	4-Aug-03	Male	Adult	5,787,065	35.54	99.03	21.2	F1-Hawaiian duck × Feral Mallard Hybrid	0.53	0.47	0.25	0.0067	30,548	23,000	PRJNA1499709	SAMN13029977
AEJR4533	Hybrid 3	Hawaii	Oahu	21.368323	−157.710297	7-Oct-03	Male	Adult	4,789,362	35.53	99.02	17.9	FX- Feral Mallard Hybrid	0.81	0.19	0.26	0.0064	37,389	200,000	PRJNA1499709	SAMN13029975
AEJR4530	Feral Hawaii mallard	Hawaii	Oahu	21.368323	−157.710297	24-May-03	Male	Adult	5,482,921	35.55	99.1	20.6	Hawaii Feral Mallard	1	0	0.2	0.0067	24,642	16,000	PRJNA1499709	SAMN13029972

## Data Availability

Illumina ddRAD-Seq reads are available in the NCBI Sequence Read Archive (SRA). The corresponding BioProject accession numbers are PRJNA991977, PRJNA1451198, and PRJNA1499709, and the BioSample accession numbers are listed in Table 1.
